# Design and analysis of 2D one-way splitter waveguide based on topological photonics

**DOI:** 10.1038/s41598-024-62816-3

**Published:** 2024-06-05

**Authors:** Mohammadreza Mehdipoura, Mohammadreza Moeini, Vahid Ahmadi, Reza Poursalehi

**Affiliations:** 1 Department of Nanotechnology Engineering, Tarbiat Modares University, Tehran, Iran; 2 Department of Electrical and Computer Engineering, Tarbiat Modares University, Tehran, Iran

**Keywords:** Photonic topological insulator, Splitter waveguides, Resonating modes, Edge modes, Optics and photonics, Optical physics

## Abstract

We present a new high-efficiency splitter waveguide design based on photonic topological insulators. The system’s robust edge states allow electromagnetic waves to propagate in the 2D waveguide without backscattering, resulting in almost 100% transmission in the outputs. We also study resonating modes in the structure and show that introducing specific defects can create such modes. We consider four domains with rods of varying magneto-optical properties to provide edge modes in the system. By eliminating rows and columns of rods, we calculate the transmission at the outputs, revealing resonating modes in the middle of the structure with spatial symmetry. Our calculations indicate that the most promising resonating mode occurs when two rods and two columns are eliminated, with a quality factor Q = 1.02 × 10^6^ at frequency f = 8.23 GHz and almost zero transmission at this frequency to the outputs. We further confirm our results using the transmission line resonator model as a semi-analytical model, which agrees well with our findings.

## Introduction

The field of topological photonics has been attracting research attention since edge state modes allow electromagnetic fields to propagate one-way without backscattering in the presence of impurities and defects^1–12^. The photonic band gaps presented in photonic crystals (PCs) have provided many applications in integrated optics. For example, magneto-optical PCs are commonly used to fabricate non-reciprocal optical circuits^13,14^. Due to their potential applications in switches and circulators, backscatter-free gyromagnetic PC waveguides have recently received significant attention. A magnetic field can easily control these waveguides to confine modes defined by edge states with group velocities directed in one direction. As a result, these systems do not have backscattering^5,15^.

There is a growing interest in utilizing topological photonic systems to develop optical splitters and routing devices, employing the backscattering-immune nature of topological edge states^16–21^. For example, Makwana et al. and Zhang et al. have developed a topological beam-splitter using valley photonic crystals, resulting in improved transmission^22–24^.In this paper, we present a novel magneto-optical splitter waveguide design that integrates topologically protected transport with resonant mode engineering to achieve high transmission efficiency across a broad bandwidth. By tailoring defects in a 2D topological photonic crystal and applying a DC magnetic field to break time-reversal symmetry, we create a T-shaped waveguide structure that supports one-way propagation of edge states and can be efficiently split into the output ports. Additionally, the application of the magnetic field allows for enhanced control and functionalities, such as optical switching. This capability enables us to switch the structure between the topological phase and trivial phase as needed, distinguishing these structures from those based on Valleytronics. There are several approaches available for analyzing defect modes and modes profiles in photonic band gap materials^15,25–28^, which are divided into two categories. Rigorous numerical methods such as finite difference time domain (FDTD) or finite element method (FEM) and semi-analytical approaches, usually refer to the expansion of electromagnetic fields in terms of their functions. The latter method has been reported to be accurate and less computationally intensive than FDTD and FEM. One of the successful semi-analytical methods to describe PC cavities and their properties, namely their resonance frequency together with their quality factor has been reported by^29^. This approach utilizes the principle of impedance matching in a transmission line to examine the flaws in PCs. In this method, the defect region is substituted with a transmission line that possesses the electromagnetic wave characteristics of a PC waveguide. This waveguide is constructed by removing a line of rods and repeating the defect region periodically along the planar axes of the PC. The transmission line breaks at both ends with an impedance, forming a transmission line resonator. The resonance frequency is calculated by determining the reflection from the PC located next to the defect zone.

## Theoretical analysis

In this paper, we propose a one-way T-shaped splitter waveguide with a tunable resonator at the cross, using the magneto-optical properties of the rods and the tailored defects to control the light, resulting from the coupling of one-way edge modes and the defect modes. Due to the topologically protected edge modes of this splitter waveguide, unlike other PC splitters studied before, the propagation of light has no backscattering, resulting in a near 100% transmission in the outputs. This design is very promising for constructing one-way magneto-optic switches. All the results are simulated by a finite element method analysis. We then compare our results from the FEM analysis with those obtained from a semi-analytical model to confirm the characteristics of the resonating modes that appear at the cross.

**Figure 1:**
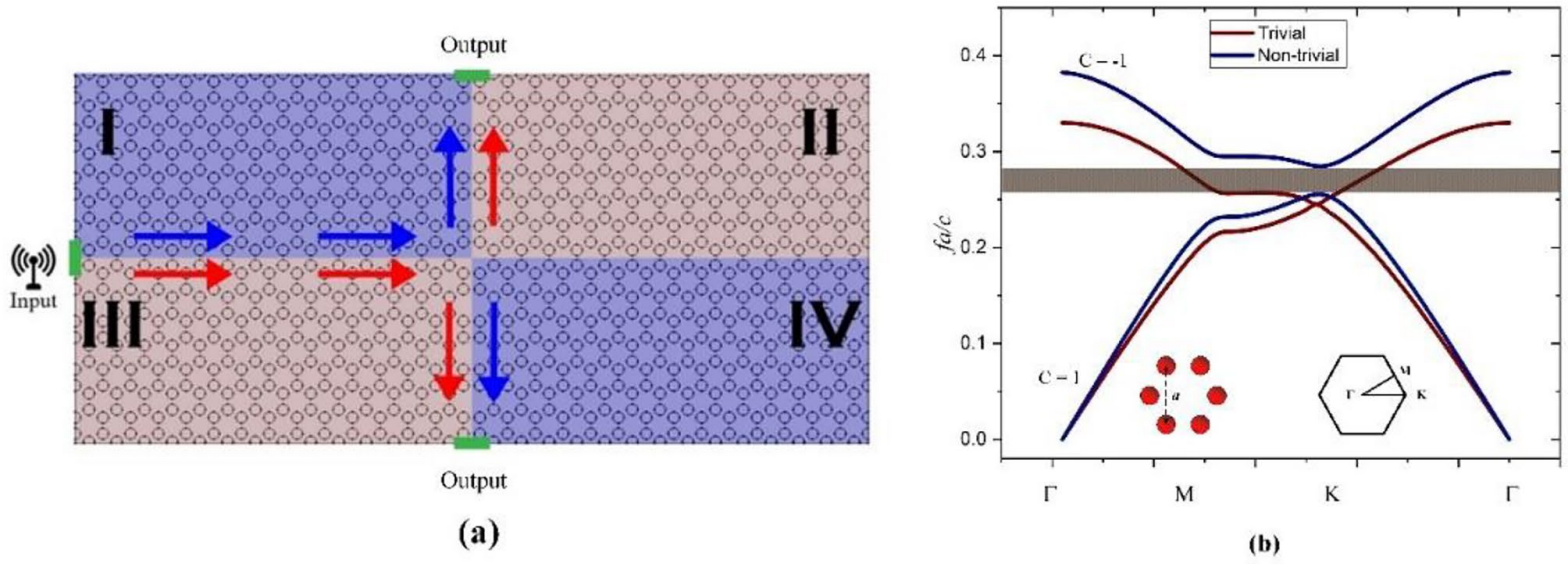
(**a**) A honeycomb structure of ferrite rods in the air with a radius of 0.2*a*. Each domain has different magnetic properties. Band diagram of the honeycomb structure. (**b**) Time reversal symmetry is broken in the band diagram by applying the magnetic field, leading to one-way edge states inside the bandgap. C represents the Chern number of the bands after the symmetry has been broken.

To make a T-shaped splitter waveguide we propose a structure with four domains as shown in Fig. 1a, where domains II and III have mirrored off-diagonal elements in their permeability tensor with respect to domains I and IV. Each domain consists of a honeycomb lattice of ferrite rods in the air with a specific permeability tensor. Each circle in the figure represents a rod with a radius of 0.2*a*, where *a* is the lattice constant. The permittivity of each ferrite rod ε is set to ε = 15ε_0_. By applying an external magnetic field perpendicular to the PC plane, we induce gyromagnetic anisotropy. The permeability tensor of the magnetic rods is given by

1 $$\mu =\left[\begin{array}{ccc}\mu & -i\kappa & 0\\ i\kappa & \mu & 0\\ 0& 0& {\mu }_{0}\end{array}\right]$$

For an applied magnetic field of 0.05 T in the z-direction, the values of μ and κ will be equal to 0.850 and 0.680, respectively^30^. The effect of material dispersion is neglected.

The band structure of the proposed structure is calculated using the conventional band theory of PCs. Considering the Maxwell's equations, we have:

2 $$\nabla \times \left({\mu }^{-1}\left(r\right)\nabla \times E\right)= \varepsilon (r){\omega }^{2}E$$

The permeability tensor μ(r) is a function of position, and ω is the frequency of the modes. We can rewrite (2) in the form of:

3 $$\langle E_{1} {|}E_{2} \rangle = { }\smallint d^{2} r{ }\varepsilon \left( r \right)E_{1}^{*} \cdot E_{2}$$

In order to have one-way edge states, one must break the time-reversal symmetry of the magneto-optical PC modes which directly affects the topological properties of the bands of the system. This effect is generally defined by the Chern number, previously studied in the quantum Hall effect extensively^31–33^. The Chern number of the n-th band is defined as:

4 $$C_{n} = 1/2\pi i\int\limits_{BZ} {d^{2} k} \left( {\frac{{\partial A_{y}^{nn} }}{{\partial k_{x} }} - \frac{{\partial A_{y}^{nn} }}{{\partial k_{x} }}} \right)$$

The integral in (4) is over the first Brillouin zone and A^nn^ is the Berry connection defined as:

5 $$A_{{}}^{nn} \left( k \right) = \langle E_{nk} {|}\nabla_{k} {|}E_{nk} \rangle$$

which is performed over the unit cell. Here, E_nk_ is the eigenmode of the n-th band.

Each energy band in a photonic crystal is characterized by a topological invariant called the Chern number. Chern number is always zero when time-reversal symmetry is preserved, as given by Eq. (4). However, by adiabatically tuning the system's permeability tensor and its Hamiltonian, one can introduce an applied magnetic field that modifies the Chern number.

Applying a magnetic field breaks time-reversal symmetry, lifting the band degeneracy and creating a bandgap. To identify topological edge states, we must demonstrate a non-zero difference between the Chern numbers of the upper and lower bands, indicating a topological bandgap. This change in Chern number upon lifting the degeneracy is denoted as ΔC = C_2_ – C_1_, where C_2_ and C_1_ are the Chern numbers of the upper and lower bands, respectively. Figure 1b shows that the magnetic field breaks time-reversal symmetry, creating a bandgap with distinct Chern numbers for each band.

Using the FDTD method^34,35^, we calculate the Chern numbers before and after applying the magnetic field to confirm the trivial and non-trivial phases. Initially, with time-reversal symmetry preserved, the Chern numbers are zero for both bands, indicating a trivial phase with a degenerate point at the K-point, allowing light propagation. However, upon applying the magnetic field, time-reversal symmetry is broken, and the degeneracy at the K-point is lifted, generating a bandgap as shown in Fig. 1b. To verify the topological nature of this gap, we calculate the Chern numbers for the first and second bands. For domains I and IV, the first band has C_1_ = 1, and the second band has C_2_ = − 1. Thus, the difference ΔC = C_2_ − C_1_ = 2, confirming a non-trivial topological gap. Domains II and III exhibit the same Chern numbers but with opposite signs. In the proposed structure, since each domain has specific topologically protected states that guide the light in the desired direction, a T-shaped splitter function can be achieved with backscatter-free properties. It has been shown ^2^ that the edge modes with the same group velocity sign in the whole Brillouin zone result in a one-way propagation on the outer boundaries of each domain. While domains II and III can guide the light clockwise on their boundaries, domains I and IV support a counter-clockwise propagation on their boundaries.

**Figure 2:**
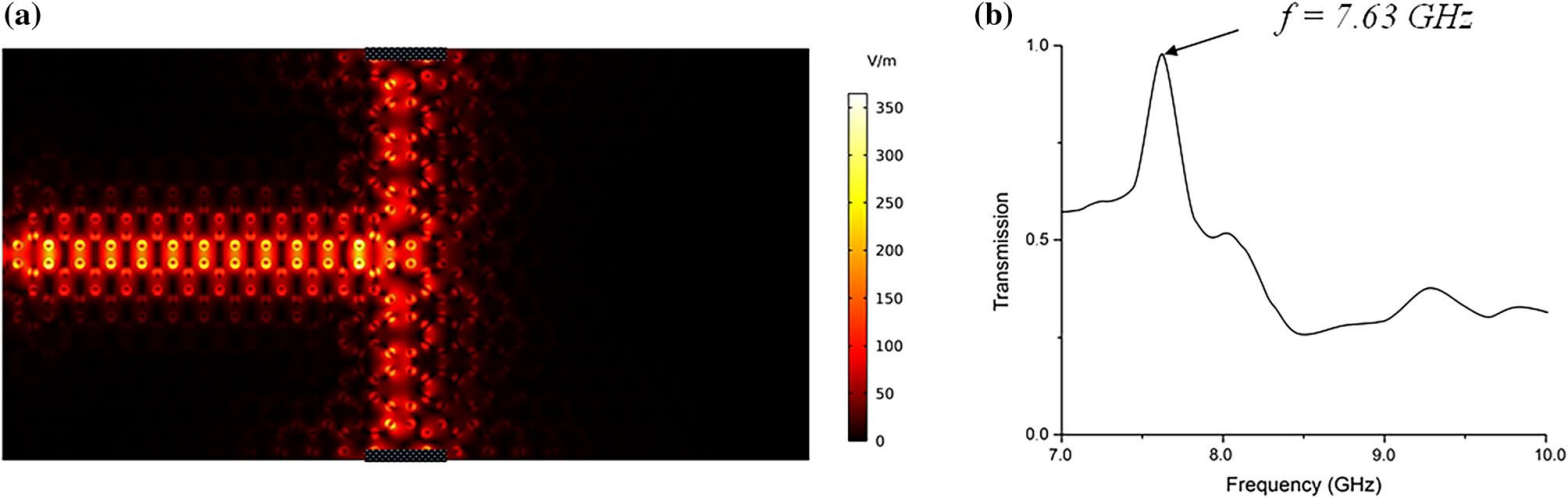
(**a**) The structure consists of four domains with no structural defects. By applying a point source in the system, the power is split to ports 1 and 2 and no backscattering is evident. (**b**) The total transmission received at ports 1 and 2 is near 100% at f = 7.63 GHz.

In Fig. 2a, we show the electromagnetic field propagation in the proposed structure based on the topologically protected edge modes discussed above. The whole structure is considered with no structural defects, however as shown in Fig. 2a, the entire PC is divided into 4 domains with different anisotropic magneto-optic properties. The interface between these domains can be used as a waveguide, due to the one-way propagation properties of the edge states of each domain. When the operating frequency is inside the bandgap, light can only propagate in the direction provided by the edge modes in the PC band structure, wherein this case as shown in Fig. 2a, light propagates only to the right as no backscattering is allowed by the edge modes and its power is split in half to reach ports 1 and 2. Here, the bandgap of the structure is non-trivial in the frequency range 7.23–7.81 GHz with edge states inside the bandgap. Based on the Chern number of the band calculated in the first Brillouin zone, the formed gap in Fig. 1 is a non-trivial topological band gap. Thus, the electromagnetic waves with frequencies inside this gap are topologically protected by the edge modes and are robust against backscattering.

To show the one-way propagation of the electromagnetic field in the waveguide, a point source is employed in the structure as depicted in Fig. 2a. Since the edge state allows only the light to move to the right side of the structure (on the interface of domains I and III), no backscattering is allowed. Also, at the right side of the structure (domains II and IV), since the edge modes allow only the propagation of electromagnetic waves to the left, there is no propagation to the right port, hence, the propagating field is split to the top and bottom ports where the edge modes exist and guide the electromagnetic waves. As shown in Fig. 2b, the total transmission power received at ports 1 and 2 is calculated. It can be seen that the total transmitted power is near 100% at f = 7.63 GHz which confirms that no backscattering is present in the system at this frequency. It is noteworthy to mention that the power received at port 3 is zero at this frequency, which is due to edge modes of the system on the interface of domains II and IV where no propagation is allowed to the right and only propagation of light to the left is possible.

**Figure 3:**
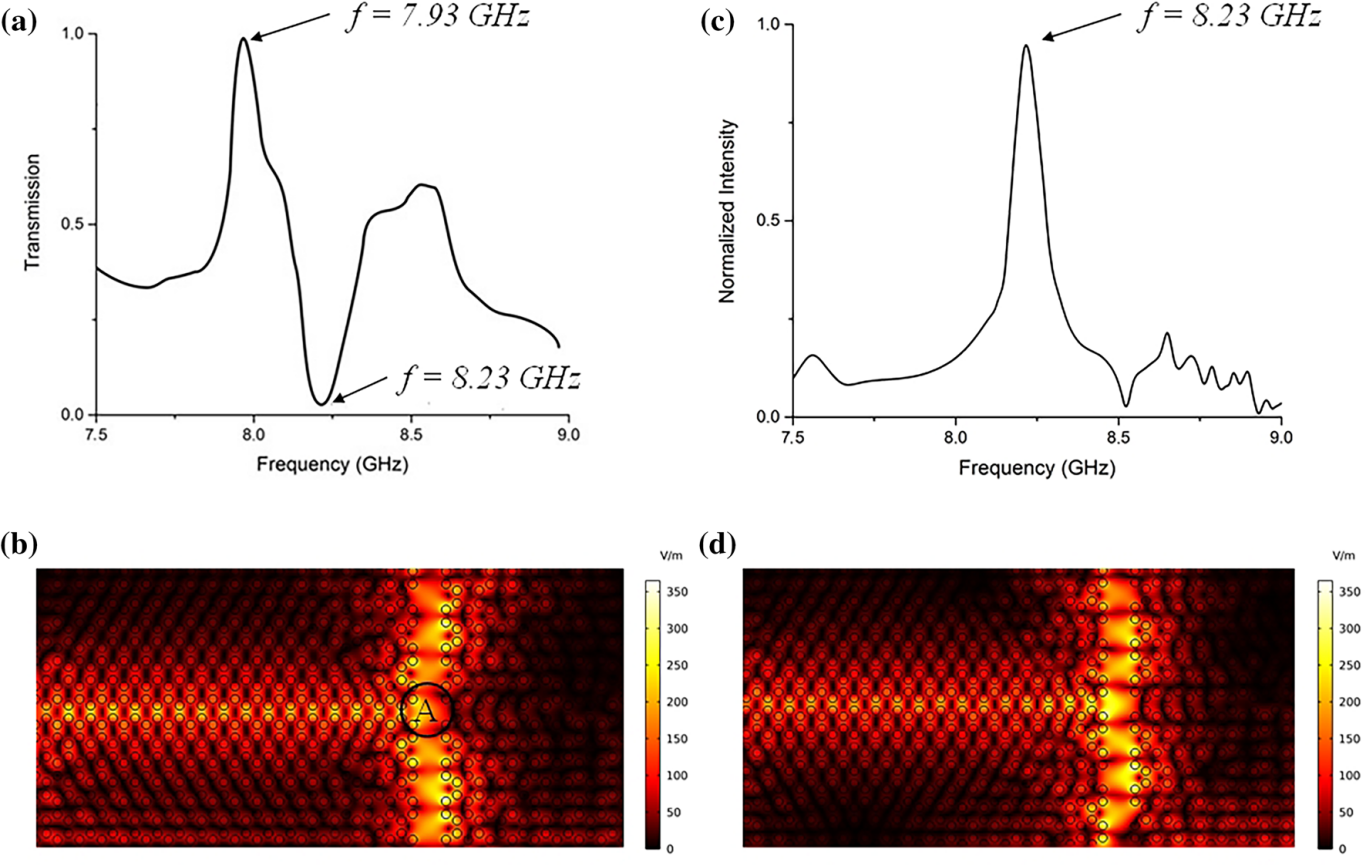
By eliminating 2 columns in the middle, a splitter waveguide is formed. (**a**) Total transmission spectrum received at top and bottom ports. (**b**) The electric field profile at f = 7.93 GHz is split to the top and bottom ports. (**c**) The normalized electric field intensity in the cavity formed at point A at different frequencies. (**d**) The electric field profile at frequency 8.23 GHz shows the guided electromagnetic field has been resonated.

Furthermore, we introduce several line defects to form waveguides with different widths at the interface of each domain. In the first study by eliminating 2 columns in the middle, a splitter waveguide is formed. The total transmission spectrum at the top and bottom ports is calculated as shown in Fig. 3a. Highly efficient transmission is achieved at the frequency of 7.93 GHz Fig. 3c shows the normalized electric field distribution. It can be seen that the electromagnetic wave is propagated from the input along with the interface of the two domains discussed above, and split to the top and bottom ports equally at point A. At 8.23 GHz a drop in the transmission spectrum is evident which shows almost zero transmission to the top and bottom ports. In Fig. 3c, the normalized intensity spectrum is shown at point A. It can be seen that at this frequency the electric field intensity at the center is increased dramatically Fig. 3d shows the localization of light formed in the cavity at point A at f = 8.23 GHz with a Q factor of Q = 6.85 × 10^5^.

**Figure 4:**
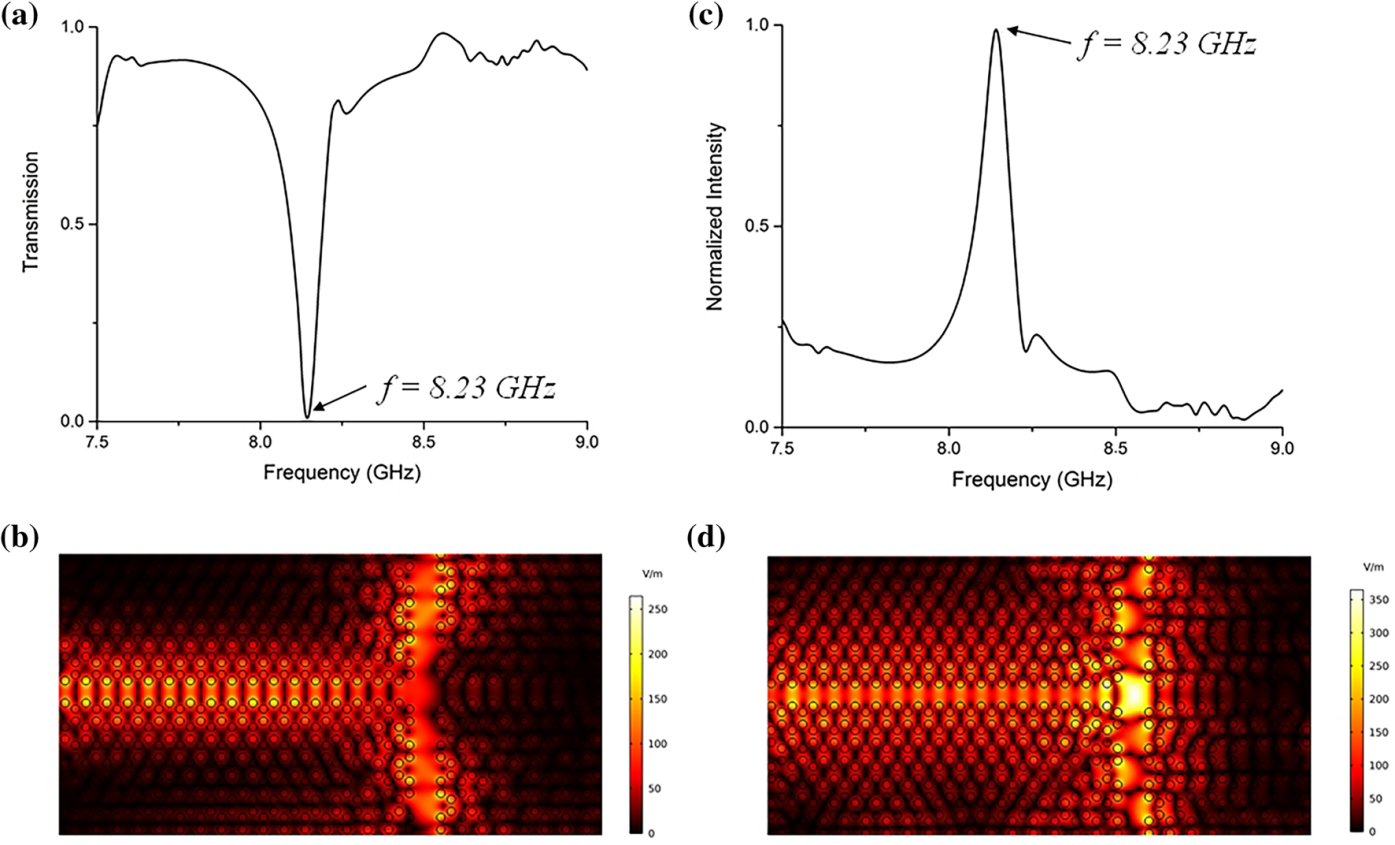
By elimination of one row and two columns at the interface of the domains, a T-shaped waveguide splitter can be formed which can guide almost all of the frequencies in the bandgap with a very well efficiency. (**a**) A huge drop at f = 8.23 GHz is seen in the transmission spectrum at (**b**) exactly at the same frequency the electric field intensity increases dramatically at the junction. (**c**) The electric field profile at f = 7.82 GHz shows that light in the waveguide is split to the top and bottom ports and (**d**) at f = 8.23 GHz the light is localized and resonated.

To investigate further, we investigate the structure where one row and two columns of rods are eliminated at the interface of the domains to form a T-shaped splitter waveguide. In this setup, almost all the frequencies in the range depicted in Fig. 4a have a good efficiency to guide the electromagnetic waves to the top and bottom ports although not all of them are topologically protected with a nonzero Chern number. However, a huge drop in the transmission spectrum can be seen at f = 8.23 GHz which confirms the peak at the same frequency in the electric field intensity spectrum Fig. 4b. The light is localized at the junction. Figure 4d shows a strong electric field intensity at the junction. The Q-factor of the resonating mode is calculated as Q = 1.02 × 10^6^.

**Figure 5:**
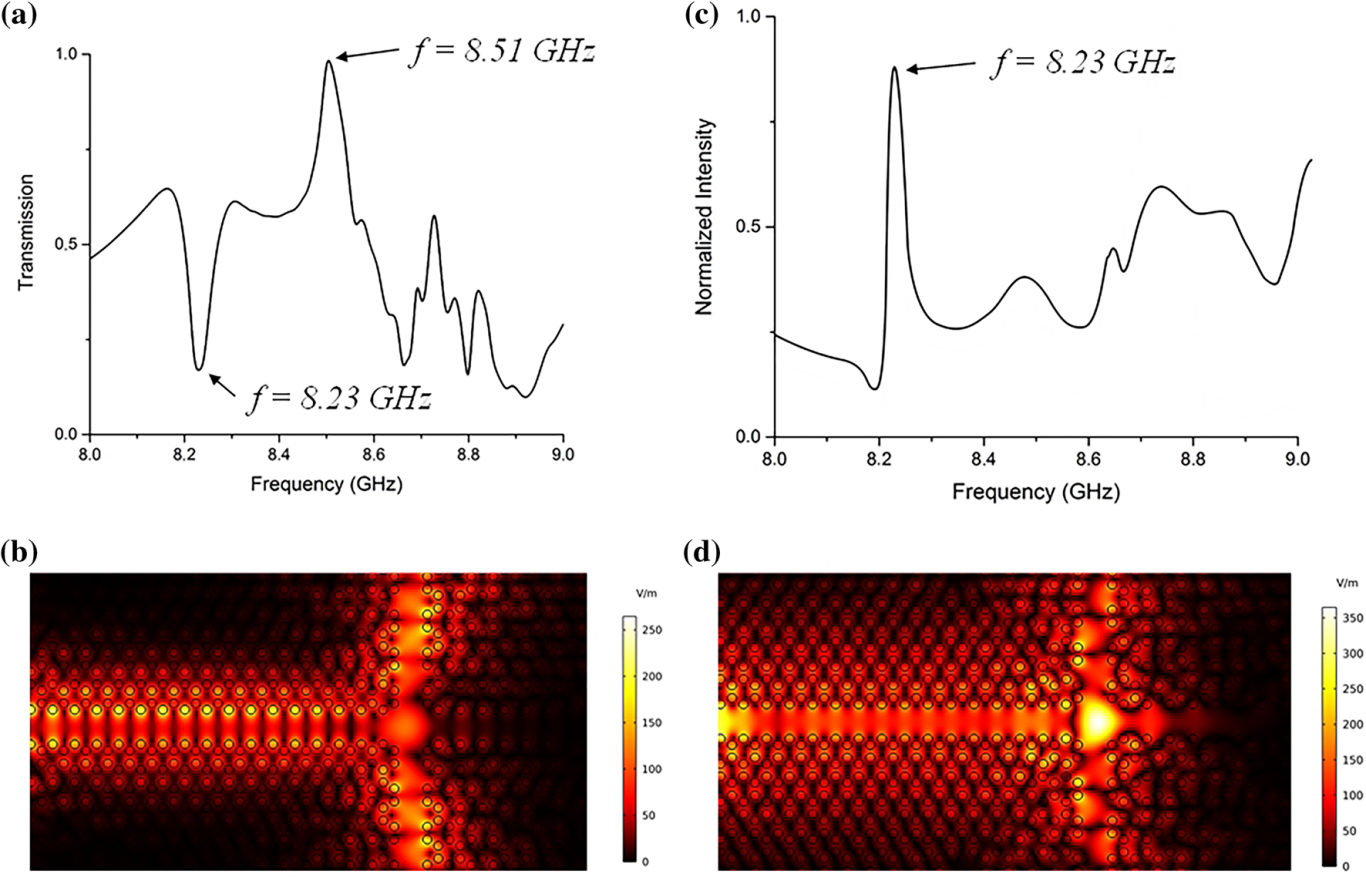
A T-shaped splitter waveguide by eliminating two rows and two columns of rods at the interface. (**a**) The transmission spectrum shows almost 100% total transmission to the top and bottom ports at f = 8.51 GHz. (**b**) The electric field intensity spectrum shows a peak at 8.23 GHz. (**c**) The electric field profile at f = 8.51 GHz, and (**d**) f = 8.23 GHz.

In the final case, the elimination of two rows and columns at the interface is studied. The total transmission at the top and bottom ports is nearly 100% at 8.51 GHz, which shows a loss-free propagation of electromagnetic field through the splitter waveguide. Figure 5c shows the electromagnetic field profile propagating from the input to the top and bottom ports. An interesting point is that although there are no physical barriers on the right side (between domains II and IV), due to the protected edge states which support the one-way propagation nature of such states, no light propagates to the right and a T-shaped splitter with almost 100% efficiency is formed. It seems that domains II and IV are acting as mirrors in the desired frequency.

In the electric field intensity spectrum shown in Fig. 5b, the peak at f = 8.23 GHz corresponds to the localization of light at the junction. It is also seen in Fig. 5a that at the same frequency, the total transmission drops dramatically. Figure 5d shows the resonated electric field profile at the junction. The quality factor of the resonated mode is calculated as Q = 1.02 × 10^7^.

**Figure 6:**
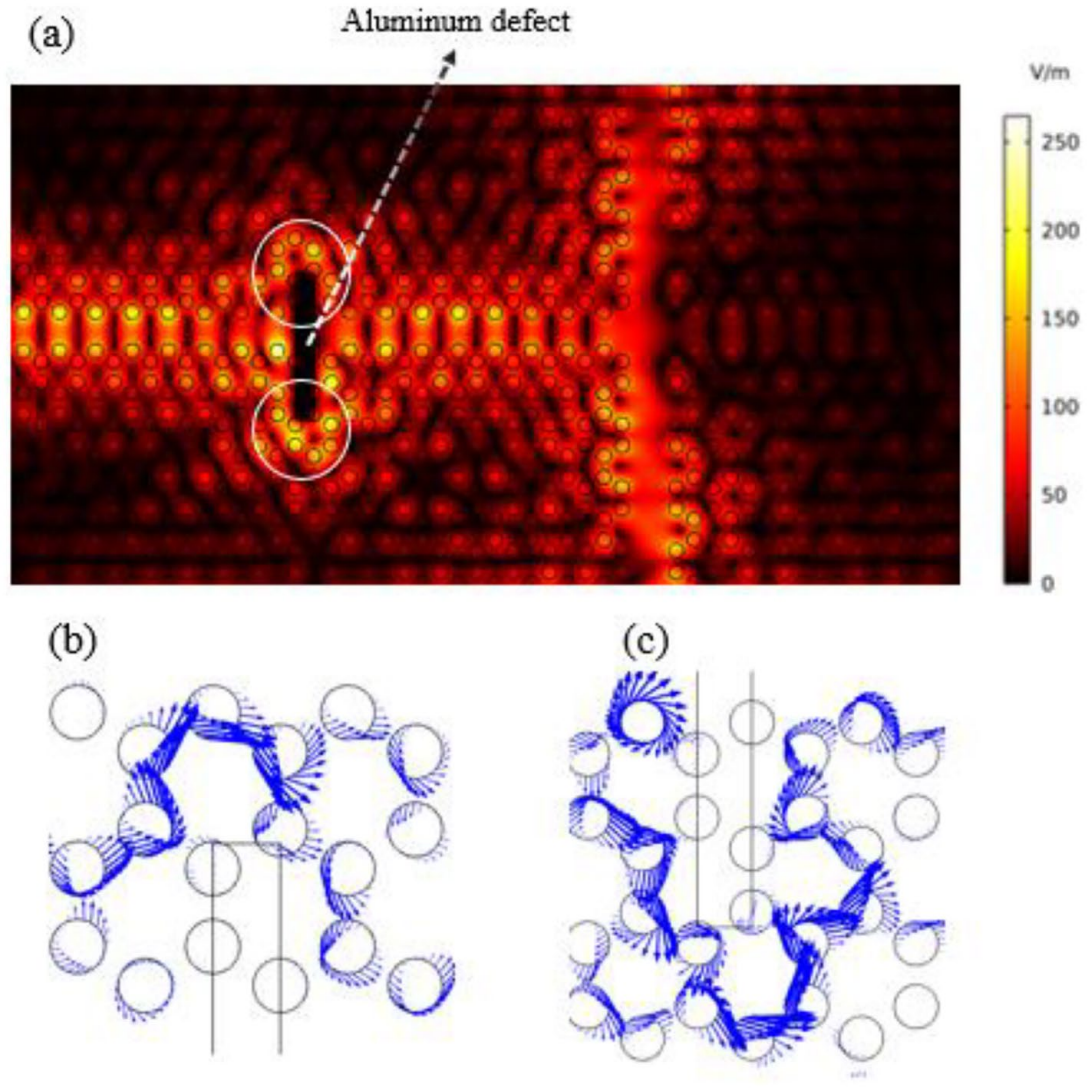
(**a**) By introducing an aluminum defect, the electric field propagates from the left to the right, around the defect through the edge states, confirming topological protection. Blue arrows represent Poynting vectors, (**b**) and (**c**) illustrate the energy propagation at the top, and the bottom of the defect, respectively.

The edge states in this setup are topologically protected, making them resistant to disruptions. To illustrate this robustness, we introduce an aluminum defect into the structure. As shown in Fig. 6a, the electric field passes around the defect via the edge states. Figure 6b and c depict the propagation of power flux indicated by the blue arrows representing the Poynting vector, equivalent to the energy propagation, at the top, and the bottom of the defect, respectively.

**Figure 7:**
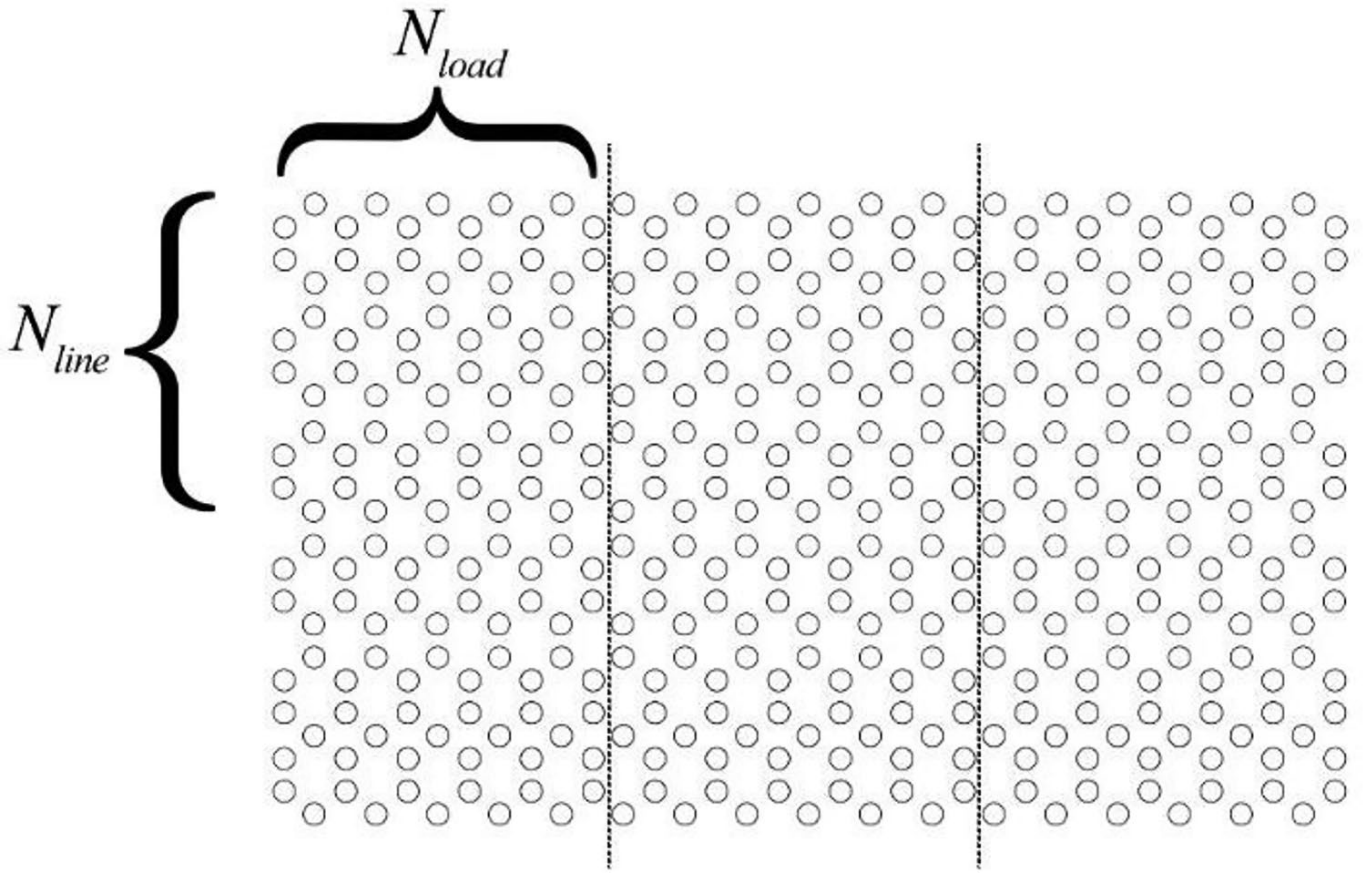
The general structure used for the analysis of the load impedance.

To confirm our results from the FEM analysis, a transmission line is considered to model the defect region whose properties are denoted by Z_c_ as characteristic impedance, β as propagation constant, and l as the length. Here the propagation constant is the same as the PC itself. The structure is created by the periodic repetition of the defect region along the planar axes. To prevent the structure from being lossy, we consider the upper and lower rows of the structure denoted by N_line_ to be large enough (Fig. 7). We have seen that for N_line_ > 8 the loss is considerably low, and thus the propagation constant is given by:

6 $$\beta \left(\omega \right)={\beta }_{r}\left(\omega \right)-j{\beta }_{i}\left(\omega \right)$$

no longer has the imaginary part. This also confirms the fact that topologically protected modes on the left side of the structure only provide positive group velocity of electromagnetic fields on the left side of the structure and ensure a total reflection on the right side of the structure, where only negative group velocity is present. The imaginary part of (6) contributes is decreasing the Q-factor of the cavity. The propagation constant of the waveguide is calculated by the planar expansion method previously reported by various papers. By calculating the reflection coefficient from the PC, it is possible to determine the normalized load impedance by:

7 $$\overline{{Z }_{L}}=\frac{{Z}_{L}(\omega )}{{Z}_{C}}=\frac{1+R(\omega )}{1-R(\omega )}$$

where R(ω) is the reflection coefficient of the incident plane wave for the propagation constant β. Each layer of the simulation consists of N_load_ columns of rods and is fully periodic along the x-axis. Since the layers on the right side of the structure have no back-propagating states for the electromagnetic waves, total reflection is achieved, resulting in a pure imaginary term for the normalized impedance. Hence, the Q factor would not decrease due to the non-existence of resistive terms.

**Figure 8:**
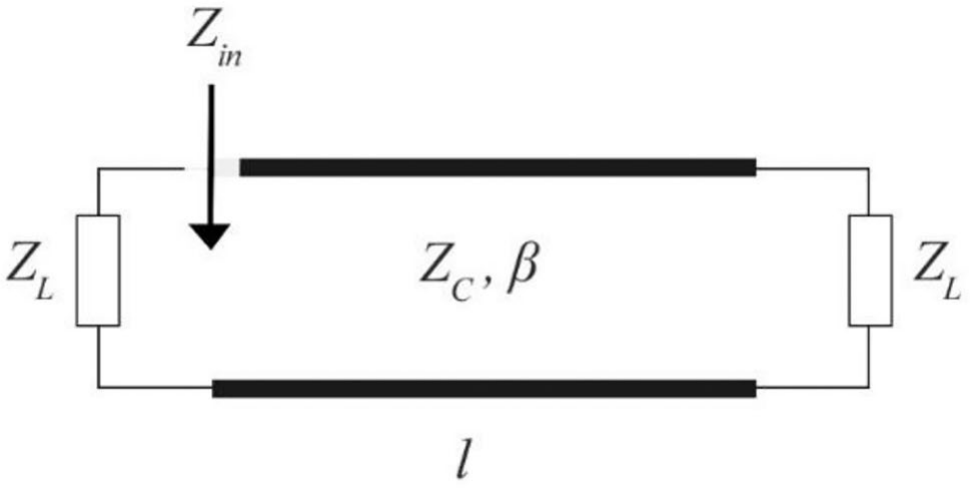
Schematic of the transmission line resonator model.

By using the model proposed by^27^ (Fig. 8) the normalized input impedance is given by:

8 $$\overline{{\text{Z} }_{\text{in}}}(\upomega )=\frac{{\text{Z}}_{\text{L}}\left(\upomega \right)+\text{tan}(\upbeta \left(\upomega \right)\text{l})}{1+{\text{Z}}_{\text{L}}\left(\upomega \right)+\text{tan}(\upbeta \left(\upomega \right)\text{l})}$$

When the complex conjugate of the normalized load impedance and the imaginary part of the normalized input impedance match, the resonance frequency is ω0 is calculated. At the resonance frequency ω_0_, the half-power bandwidth of the total impedance is then extracted to find the quality factor of the resonator as:

9 $$Q=\frac{{\omega }_{0}}{BW}$$

**Figure 9:**
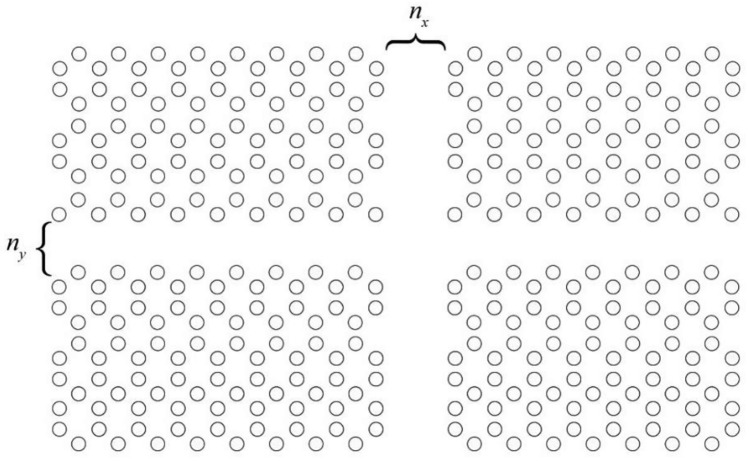
Number of rows and columns eliminated in the transmission line resonator model denoted by n_x_ and n_y_.

By calculating the propagation constant of the transmission line and the normalized impedance using Eqs. (6) and (7) we extract the resonance frequency (8) and quality factor (9) of each setup and compare the results with that of our FEM analysis. We introduce different defects to the transmission line resonator model by varying n_x_ and n_y_ as the number of rows and columns removed to form a resonator as depicted in Fig. 9.

Table 1 shows the results obtained from the calculations we made using both the FEM method and the semi-analytical approach of the transmission line resonator model. The results are not the same, however, a little deviation is expected since the calculation parameters differ in each case. The calculation time for the transmission line resonator model is significantly lower than that of FEM, and since the deviation in results is quite little, it can be neglected.

**Table 1:** Comparison of the resonant frequency and Q-factor of different cavities in the proposed structure calculated by both FEM and semi-analytic approaches.

*n*_*x*_ × *n*_*y*_	Resonating frequency (GHz)		Q-factor	
	FEM	Semi-analytic	FEM	Semi-analytic
2 × 0	8.2314	8.2319	1.02 × 10^6^	$1.13\times {10}^{6}$
2 × 1	8.2318	8.2381	6.85 × 10^5^	$6.92\times {10}^{5}$
2 × 2	8.2315	8.2298	1.02 × 10^7^	$1.12\times {10}^{7}$

## Conclusion

In conclusion, we have proposed a gyromagnetic waveguide splitter capable of light propagation without backscattering. The edge modes guide the light from the left port to the right, splitting it to the top and bottom ports at point A. By introducing line defects in the structure, we demonstrated that opposing domains act as a mirror, allowing certain modes to resonate at point A due to the one-way propagation of electromagnetic waves. Our calculations revealed that removing two columns in the middle resulted in a resonating mode at f = 8.23 GHz at point A, while a robust topologically protected edge state at f = 7.93 GHz achieved near 100% transmission to the top and bottom ports. Removing two rows and two columns did not shift the resonating frequency, but at f = 8.51 GHz, nearly 100% transmission was achieved. Additionally, eliminating two rows and two columns in the domain interface maintained the resonating mode at f = 8.23 GHz, with high transmission to the ports across most frequencies. Additionally, by removing two rows and two columns it was shown that the resonating frequency was not shifted as the distance between the domains in the right and the domains in the left were unchanged. However, at f = 8.51 a near 100% transmission to the top and bottom ports was achieved. Finally, by eliminating two rows and two columns in the interface of the domains, it can be seen that the resonating mode at f = 8.23 GHz is unchanged but the transmission to the ports is high in most frequencies of the studied range. Coupling of the edge modes by tailoring the distance between the top and bottom domains results in a change in the guided modes in the waveguide. However, by changing the distance between domains in the left and right resonating modes can appear in such a system. Our calculations indicate that the best quality factor refers to the cavity formed by eliminating two rows and two columns in the structure at f = 8.23 GHz. Furthermore, the system designed above can also be used as an on/off switch by controlling the applied magnetic field. To confirm our calculations, we used a semi-analytical approach to confirm the frequencies that resonate at the cross in the structure. We showed that both approaches lead to the same resonating frequencies with high Q-factors.

## Data Availability

The datasets used and/or analyzed during the current study are available from the corresponding author upon reasonable request.
